# The Turning Point of China’s Rural Public Health during the Cultural Revolution Period: Barefoot Doctors: A Narrative

**Published:** 2018-07

**Authors:** Youngsub LEE, Hyoungsup KIM

**Affiliations:** 1. Asia Contents Institute, Konkuk University, 120 Neungdong-ro, Gwangjin-gu, Seoul, Republic of Korea; 2. College of Humanities, Chung-Ang University, 84 Heukseok-ro, Dongjak-gu, Seoul, Republic of Korea

**Keywords:** Barefoot doctor, Cultural revolution, Medical insurance scheme, Primary health care

## Abstract

**Background:**

According to Mao Zedong’s orders, the Communist Party of China made efforts to perform the system of ‘barefoot doctors’ even during the political mayhem of the Cultural Revolution. This pioneering medical system made a great contribution to medical services for rural communities and the public health system from 1960s to 1970s. Attracting new attention in the beginning of the 21st century, the barefoot doctor system influenced the formation of the Chinese medical system of unique structure.

**Methods:**

Utilizing and analyzing the currently existing research outcomes on ‘barefoot doctors’, we investigated the two overlooked characteristics in the Chinese medical system originated from the barefoot doctor system; i) why the barefoot doctor system attracts new attention in the 21^st^ century and ii) why and how Western and Chinese medicine could systematically be combined, which is the unique phenomenon in the world.

**Results:**

The barefoot doctor system satisfied the Chinese government’s political aims and realistic request under the banner of Cultural Revolution simultaneously. In reality, this system gratified prevention-oriented modernized public health policy, reducing serious gap of medical services between cities and rural areas. Yet, this leading system was abolished in 1980s without timely use.

**Conclusion:**

In the present, the barefoot doctor system is holding limelight again as a successful precedent to intensify preventive public health service all over China, especially for underdeveloped areas. Moreover, Chinese medicine-friendly stance to utilize ‘doctors of Chinese medicine’, absolute majority over those of Western medicine, created the uniqueness of integrative medicine.

## Introduction

The Qing Dynasty launched the Department of Health within the Police Agency under the Ministry of Police in 1905. The Department of Health was the first organization that took the responsibility for a series of health-related work, such as the establishment of medical schools, controls over medical doctor’s license, prevention against epidemic, and so on (1).

After the Xinhai Revolution that terminated the dynastic system in China, the Chinese Nationalist Party endeavored to build up modernized western-style health service in the area under its direct control. This procedure was a step to establish preventive public health system, but not to treat disease. Realizing the limit of traditional Chinese medicine to effectively control various diseases, the Chinese Nationalist Party tried to make various efforts to set up western modernized public health service and medical education near 1920. Westernized Department of Public Health Service was opened within the Ministry of Domestic Affairs of China in 1927. Then the Department of Public Health Service became a central office. It became the Ministry of Public Health Service in 1945.

After Mao Zedong proclaimed the beginning of the People’s Republic of China at Tiananmen Square on October 1^st^ of 1949, the communist government began to modernize public health service incessantly. The government of the communist party leaned on the Soviet Union’s system and support that differed from those of the USA. Up to 1965, there existed more than 230 educational institutions to educate medical professionals of the western medicine. The total number of such medical professionals was over 200,000. Under the regime of the Chinese Nationalist Party, the Ministry of Education initiated the national Peking medical school (now, Peking University Health Science Center) in 1912 as a leading professional medical school of western medicine. In comparison with 1912, the launching year of the medical school, the number of freshmen was only 72. This number was multiplied at least more than 2,700 times for 50 years. However, considering the vast land and the largest population in China, 200, 000 medical professionals were still very low in number. Besides, these medical schools and institutions were centralized in cities. According to the exemplary of the Soviet Union’s medical system, the communist government built up general hospitals in provinces (more than one polyclinic) in the county and moving clinics in the village. Following the Soviet Union’s characteristic that emphasized preventive health care, the communist government opened disease-prevention centers besides treatment service (2). On June 25 of 1965, one year before the outbreak of the Cultural Revolution, Mao Zedong insisted on the need to cultivate barefoot doctors to improve undeveloped rural health care service. On the very next day, he severely criticized the public health care system. To summarize, the public health care service administered by the Ministry of Health applied only 15% of the whole socialists such as revolutionists, bourgeoisie, urban residents, and so on. Consequently, the majority of people could not receive benefit from medical and public health care services (3). Mao Zedong pointed out a series of problems of public health care policy which included prevention-oriented public health service (but not treatment-oriented medical service), equal medical-education-opportunities open to everyone irrespective of his or her education level, reduction of education period to less than three years, and supply of trained medical personnel to rural areas. These claims on public health care service by Mao were applied into practice. Indeed, the period of medical training was curtailed. As a result, they invigorated preventive medical service rather than treatment-based service. Barefoot doctors were the ones who had reformed the public health care system over underdeveloped rural areas for the purpose of prevention.

This research investigated the reason why barefoot doctor system has been newly spotlighted in the 21st century, and the unique Chinese and western integrative medicine, emphasizing the formation of barefoot doctor system, the role of Chinese medicine inside the system, and its impact on the Chinese medical system.

## Methods

There already exist many general research on ‘barefoot doctors’ (4, 5). This paper examines the currently unappreciated and overlooked characteristics that originated from the barefoot doctor system by way of analyzing and utilizing existing research outcomes on it.

- The reason why the barefoot doctor system resumes attracting new attention: The systematic Chinese Medical Reformation of 1985 formally abolished the title of ‘barefoot doctors’, stopping the system from being in use. It is universally recognized that revolutionary changes of the social structure make it impossible to put it into practice. Nevertheless, this research explores the reason why the barefoot doctor system has attracted new attention in the 21^st^ century within the structural characteristics of the establishing process of the barefoot doctor system.
- The interrelationship between the barefoot doctor system and the Integrative Medicine model: This research not only probes public awareness and changes of legal status of the traditional Chinese medicine in the process of modernization of China, but also determines why and how Chinese Integrative Medicine emerged, which is the unique and different from that in Korea and Japan.

## Results

### Characteristic of the Emergence of Barefoot Doctors

The barefoot doctor system was operated for residents outside urban areas and rural areas, especially in the countryside. For rural residents, the Rural Cooperative Medical Insurance Scheme (RCMIS) existed together with the barefoot doctor system.

Having established the People’s Republic of China in 1949, the communist government founded the public health care system that covered many areas ranging from big cities to small villages, including remote rural areas. From a perspective of medical insurance system, almost all citizens acquired effective benefits from this insurance system. Government workers, university students, and wounded veterans were covered by Government Employee Insurance Scheme (GEIS). Urban workers of government-owned companies, retirees, and his or her dependents were covered by Labor Insurance System (LIS). Farmers were covered by Rural Cooperative Medical Insurance Scheme (henceforth, RCMIS). RCMIS is a sort of social insurance operated by the fund that farmers and their villages contributed jointly. The origin of this insurance was ‘mutual help tradition’ which came into being spontaneously in some rural areas in 1940s (6).

The population of China was over five hundred million in 1949. More than 90% of them were involved in agriculture. RCMIS was a system for these farmers in rural communities. Through this medical system supported by the central government, farmers and their villages who had not benefited from well-organized medical service could secure labor force, facilities, equipment, medicine, medical supplies, and so on required for preventive public health care service and primary health care with subsidies from the central government and joint-investment of farmers and their cooperation. RCMIS was started in Shanxi province, Gansu province, and Níngxià province in the mid of 1950s. It kept expanding to other agricultural areas such as Hebei province, Henan province, and so forth. As a result, RCMIS of “prevention-primary, treatment-subsidiary” showed outstanding effectiveness all over China (7). Finally, the central government began to pay attention to RCMIS. In 1956, the Ministry of Health approved of its excellent effect and started to spread it all over the country. In 1965, RCMIS was initiated in 10 provinces, including Hubei, Jianxi, Jiangsu, Fujian, Guangdong, and so on, and many autonomous rural communities, including Uygur autonomous region of Xinjiang province (8).

What was significant was that RCMIS was operated mainly by the joint-fund that farmers and agricultural communities raised spontaneously despite subsidy from the central government. It was found to be an autonomous and independent system in the countryside. Moreover, the barefoot doctor system played an important role in providing ‘labor force’ for RCMIS. As mentioned above, with Mao’s order, the system of ‘barefoot doctors’ came into practice over the whole country. During the Cultural Revolution, the barefoot doctor system was regularized in an earnest way. Barefoot doctors were not authentic doctors. They were just those who had been trained in a short term (varying from 3 to 6 months) or from 2 to 3 years if necessary. They were in charge of public health care which was ‘prevention-oriented’ and primary health care in health centers in the countryside. Thanks to the short-term training way of barefoot doctors, medical personnel were radically multiplied in number in agricultural areas (9).

For the 10 years of the Cultural Revolution, most people’s communes and production brigades implemented RCMIS. Over 90% of famers in the country participated in RCMIS, building up public health care organizations by their own unit. Under the system of RCMIS, almost 1.4 million barefoot doctors took the responsibility for farmers’ health care service together with 5.1 hundred thousand authentic doctors and 23.6 million medical assistants. Barefoot doctors were distinct from authentic doctors not only in medical education-level, but also in social rank and status. Barefoot doctors were not government workers. They were called ‘a farmer and doctor’ (half farmer and half doctor) who had two jobs – being a doctor and farmer at the same time. They received monthly salary that was generally 50% of a regular doctor from RCMIS (10).

In 1980s, Deng Xiaoping’s economic reform policy in China triggered large-scale reconstructions of the public health care system. At that time, government-owned companies, organizations, and people’s communes in urban areas had collapsed. They could not afford to support RCMIS anymore. They shut down many medical schools and dispatched doctors to every corner of the country, which made the quality of medical service decline. During Deng Xiaoping’s regime, the government increased doctor’s training period from 1–3 years to 5–8 years, balancing portions between prevention and treatment. Later in 1985, ‘barefoot doctors’ were reorganized as ‘country doctors’ (11).

### Characteristic of Integrative Medicine within the Chinese Health Care System

Having adopted western medicine, the Chinese nationalist party government took an exclusive position against traditional Chinese medicine. They consistently implemented governmental policy to belittle traditional Chinese medicine. Therefore, they westernized all medical education and medical care system. However, the communist party acted to the contrary. They remained in unstable and poor surroundings.

Therefore, they were in favor of the traditional Chinese medicine which could be instantly applied to practical use. It was after the establishment of the People’s Republic of China that the communist party systemized public health care by absorbing western medicine. Even after the reformation of the health care system into western style, the communist party preserved the Chinese medicine as an auxiliary and supportive one.

The 1^st^ National Public Health Care Conference of 1950 proclaimed three broad principles: i) “orienting prevention,” ii) “concentrating on working people, farmers, soldiers,” and “integrating Chinese and Western medicine.” In the 2^nd^ National Public Health Care Conference of 1952, four broad principles of New Health Care of China were confirmed by adding “combining Public Health Care with public movement” to the given three principles (12). The principle of “combining Chinese medicine with Western medicine” was an endeavor to unite bifurcated attempts to modernize the Chinese medicine (or scientification) and to popularize western medicine (or sinicization). With regard to this, it was claimed that this principle prioritized the Chinese medicine. This turned out to be a good exemplary to make the best of existing doctors of Chinese medicine together doctors of western medicine who populated in urban areas (indeed, with limited numbers) and to systemize medical treatment and public health care service (13). Moreover, the communist party government was in favor of the barefoot doctor system as well as the Chinese medicine. According to Li Decheng, “‘barefoot doctors’ were selected by corresponding villages. Their training program also consisted of both western and Chinese medicine. In other words, this situation made ambiguous identity of being neither a Chinese doctor nor a western doctor, or being a Chinese doctor as well as a western doctor. ‘Barefoot doctors’ were educated in a city larger than a village. They were sent back to their own village. This easily appealed to local people’s close links and public health care service (14).”

There was another reason to educate both Chinese medicine and western medicine at the same time for effective fostering of medical professionals and their dispatching to the scenes in need. The representative reason was that medicinal herbs as effective medicine were available extemporaneously in given surroundings, considering that medical supplies were lacked at that time. “Most barefoot doctors treated patients with Chinese herbs. They played a significant role in developing and stabilizing RCMIS. Chinese herbs are easy to get and cheap or affordable to buy. Besides, the majority of rural residents have the tradition and experience of using Chinese herbs (15).”

Here are the real contents of *Barefoot Doctor Handbook* given to barefoot doctors at that time (16).

Chapter 1. How to prevent disease? / Chapter 2. How to check and treat disease? / Chapter 3. How to cure disease with Chinese medicine? / Chapter 4. Acupuncture and massage. / Chapter 5. New treatment / Chapter 6. Chinese herbs for common use / Chapter 7. Relief in war-field and Preparation for nuclear weapon, chemical weapon, and germ weapon / Chapter 8. Diagnosis of frequent symptom and treatment / Chapter 9. First aid / Chapter 10. Epidemic / Chapter 11. Parasitic disease / Chapter 12. Internal disease / Chapter 13. Children’s frequent diseases / Chapter 14. Woman’s disease and pregnancy / Chapter 15. Family plan / Chapter 16. Surgical disease / Chapter 17. Injury / Chapter 18. Eye disease / Chapter 19. Ear, nose, throat disease, and dental disease / Chapter 20. Dermatological disease / Chapter 21. Appendix

This 744-paged handbook spares 80–90 pages for Chapter 3. There are over 100 pages for Chapter 6. Chinese Herbs are abundant in its contents. Medicinal herbs of western medicine were mentioned in Chapter 21, Appendix. However, 79-paged information was introduced for western medicinal herbs, which is less than that for Chinese herbs. One can assume that western medicinal herbs must have low frequency of use because its information is only introduced in the appendix. This phenomenon can be regarded as a measure to save rural areas used to lack authentic doctors and medical supplies. For this reason, barefoot doctors thought highly of three folk resources: folk remedy, folk medicinal herbs, and folk drugs. They simultaneously put four kinds of self-sufficiency (self-planting, self-collecting, self-making, and self-using) into practice (17).

To summarize, under the Chinese communist party’s practical need, Chinese medicine prevailed in rural areas rather than in urban areas. From the beginning, the communist party often excluded Chinese tradition and culture and stigmatized them as ‘feudal’ or ‘unscientific’. However, realistic and practical need of Chinese medicine during those days led to the utilization of Chinese medicine in amity.

## Discussion

### Why the Barefoot Doctor System is Newly Obtaining Spotlight in the 21^st^ century?

According to Zhang Zikuan’s evaluation, “although they did not have high quality of medical skills, barefoot doctors played a significant role in informing the people of public health care service, developing a public health care campaign, planning to prevent epidemic, fulfilling RCMIS, and treating slight injury and disease.” In fact, barefoot doctors’ achievements resulted in outstanding changes in the level of public health care service. Infant death rate was decreased from 200 deaths per 1,000 live births in 1962 to 34 deaths per 1,000 live births in 1982, while life expectancy was increased from 35 years to 60 years (18).

Therefore, barefoot doctors working in the 1960s and 1970s made a great contribution to the foundation of rural public health care and medical service as well as rural residents’ welfare. ‘The barefoot doctor system’ made a major contribution to China. It also greatly influenced the ‘Alma-Ata Declaration’ that primary health care would be the best way to secure all humans’ health and during the joint-conference of WHO and UNICEF in Alma-Ata of the Soviet Union. It became the role model for other medically underdeveloped countries (19).

Yet, ‘the barefoot doctor system’ was obviously devastated by the termination of the Cultural Revolution. Ultimately, distribution of income and changes of social structure resulted in the replacement of ‘barefoot doctors’ by ‘county doctors’ in 1985. In principle, Chinese socialism guaranteed people’s life with labor wage under planned economy controlled by the central government. The reduction of planned economy caused by Chinese economic reform and the expansion of market economy began to crack ‘iron rice bowl’ used to be guaranteed by the government. Furthermore, economic distribution was decided by the criterion of a worker. Those who cannot work such as children and severely disabled people could only be protected if they belonged to a household where a worker served as the head of the family. These were dependents for workers to support. If workers lost their stable salary, workers’ households also began to collapse. People’s communes and cooperation also began to fall apart. Finally, the foundation where the barefoot doctor system was based on became disintegrated, consequently dissembling the barefoot doctor system. After that, it was impossible to resurrect the barefoot doctor system. Meanwhile, the outbreak of SARS in China in 2003 revealed a serious weakness in the Chinese preventive system. Especially, after the teardown of RCMIS and the barefoot doctor system, new public health care system has not been established. According to the report by WHO in 2000, China which used to be a role model for developing countries was ranked the 188^th^ of 191 countries in inequity of resource distribution for medical treatment or public health care. For this reason, China brought up the necessity of ‘the New Rural Cooperative Medical Insurance (henceforth, NRCMI)’ in 2002. In 2003, the Chinese government initiated NRCMI on a trial basis. In 2010, NRCMI was applied to countryside. Unfortunately, the barefoot doctor system used to be paired with RCMIS has not been reestablished yet (20).

One began to have interests in how RCMIS and the barefoot doctor system won success in preventive service and how they made such a big achievement in the present situation. In the 21^st^ century, resurrection of public health care system for agricultural areas is necessary.

### Interrelationship between ‘Barefoot Doctors’ and ‘Integrative Medicine’ is unique in China

After announcing ‘Integrative Medicine’ as one of four broad principles in 1950, Mao Zedong established the Chinese Academy of Chinese Medical Science (Henceforth, CACMS) in 1955 for Integrative Medicine. About 2,300 western doctors graduated from CACMS after learning Chinese medicine. These graduates were the 1^st^ generation doctors who performed integrative medicine. Later, a 10-year-plan to develop integrative medicine was announced in 1976. The plan specified clearly that each province, city, and autonomous region must establish more than one hospital of integrative medicine by 1980. Since 1980, schools of Chinese medicine and schools of western medicine have opened a total of 28 doctor’s programs and 87 master’s programs and admitted integrative medicine as a formal academic course. As a separate degree program to make doctors have opportunity to learn Chinese medicine as well as Western medicine, Western medicine coursework was offered as a regular curriculum in schools of Chinese medicine, while Chinse medicine coursework was offered in schools of Western medicine (21).

This realization and systematic support made Chinese medicine unique and distinctive from Taiwanese medicine and Chinese medicine in Japan or Korea. It had succeeded to the tradition of Chinese medicine. In the case of Taiwan, following the tradition of the Republic of China, western medicine holds a dominant position in Taiwan. This is the same case in Korea and Japan. In Korea, western medicine predominates overwhelmingly, although there is some Chinese medicine. In the case of Japan, Chinese medicine was expelled from formal medical treatment. It remained just as a folk remedy. To systemize the communist basis to combine Western medicine with Chinese medicine as well as building up coexisting system of integrative medicine in China, more than 90% of medical schools for western medicine in China opened courses for Chinese medicine, while more than 30% of medical schools for Chinese medicine launched courses for Western medicine. In Korea, Chinese medicine contains a large portion of western medicine, since the latter serves as a global standard. However, it is not the case vice versa. Most of all, China qualifies doctors of integrative medicine (doctor who studies western medicine for 5 years plus Chinese medicine for 2 years). This medical system is the uniqueness of China (22). Cooperative treatment between doctors of western medicine and doctors of Chinese medicine does exist in Korea. However, license and qualification for doctors of western medicine and Chinese medicine are strictly separated.

## Conclusion

Although the barefoot doctor system was extinct, China regarded it as a successful precedent to realize preventive health care service all over China. The Chinese government endeavors to absorb the strength of preventive health care service of that time with low-cost and high-efficiency, while reacting to social structures that are totally different from those at that time. The Chinese medicine-oriented policy to make the best of doctors of Chinese medicine, who used to predominate in number, lasted, finally forming the unique system of integrative medicine in China.

## Ethical considerations

Ethical issues (Including plagiarism, informed consent, misconduct, data fabrication and/or falsification, double publication and/or submission, redundancy, etc.) have been completely observed by the authors.
